# Countermovement Jump Kinetic Impairments in Elite Athletes Before and After ACL Injury: Force-Time Waveform Versus Discrete Kinetic Analysis

**DOI:** 10.1155/tsm2/1176787

**Published:** 2025-05-07

**Authors:** Cassidy de França, Matthew J. Jordan, Tanita Botha, Helen Bayne

**Affiliations:** ^1^Department of Physiology, Faculty of Health Sciences, University of Pretoria, Pretoria, Gauteng, South Africa; ^2^Intergrative Neuromuscular Sport Performance Lab, Faculty of Kinesiology, University of Calgary, Calgary, Alberta, Canada; ^3^Department of Statistics, Faculty of Natural and Agricultural Sciences, University of Pretoria, Pretoria, Gauteng, South Africa

**Keywords:** knee injury, muscle power, rehabilitation, return to sport, vertical jump

## Abstract

Pre-injury and post-injury countermovement jump (CMJ) force-time data were obtained for elite athletes 6 months after anterior cruciate ligament surgery (ACLR). Jump kinetics were analysed using a traditional phase-specific approach, and force-time data of the CMJ waveform were analysed using statistical parametric mapping (SPM). Elite athletes (*n* = 10; female *n* = 6, age = 22.0 ± 3.5 years, mass = 75.9 ± 11.5 kg) performed CMJ testing before (T0) and after ACLR (T1; 24 ± 3 weeks post-surgery). Differences in discrete and continuous metrics were analysed for (1) within-limb differences between T1 and T0 and (2) between-limb differences at T1 and T0. Lower involved limb propulsive impulse (T1: 6.4 ± 1.6 N∙s/kg; T0: 7.7 ± 1.4 N∙s/kg, *p* = 0.002) and peak force (T1: 6.4 ± 1.6 N/kg; T0: 7.7 ± 1.4 N/kg, *p* = 0.002) were found after ACLR compared to baseline. After ACLR (T1), lower involved limb propulsive impulse was found compared to the uninvolved limb (involved: 1.26 ± 0.54 N∙s/kg; uninvolved: 1.58 ± 0.56 N∙s/kg, *p* = 0.007). SPM analysis revealed specific within-limb force loss, notably reduced involved limb propulsion force at T1 compared to pre-injury at T0 (*p* < 0.001) between 92% and 99% of the CMJ (end of propulsion) and between 36% and 37% of the CMJ (i.e., late unweighting to braking phase transition). SPM analysis revealed within-limb CMJ force loss that was not seen with the discrete analysis, highlighting the complementary value of SPM waveform analysis alongside discrete analysis to identify neuromuscular impairments in stretch-shortening-cycle function in elite athletes after ACLR.

## 1. Introduction

Anterior cruciate ligament (ACL) rupture is a devastating injury for elite athletes [1, 2] and often requires surgical reconstruction (ACLR) followed by prolonged rehabilitation [3]. However, many athletes demonstrate persistently reduced neuromuscular function after ACLR [4, 5]. Neuromuscular deficits include decreased lower limb muscle strength [6–8] and biomechanical impairments [4, 9], along with reduced force output and increased kinetic asymmetry in vertical countermovement jump (CMJ) testing [7, 10–12].

The CMJ is a coupled eccentric-concentric movement, inherent to many athletic movements across various sports, that has frequently been used to reliably assess muscle function after ACLR [5, 10]. Also, the fact CMJ testing permits an analysis of eccentric loading upon initial deceleration, a mechanism of noncontact ACL rupture, makes it pertinent for return to sport testing protocols [10, 13]. More specifically, a kinetic analysis of the eccentric braking and concentric CMJ phases has been used to assess mechanical muscle function after ACLR [6, 8, 10, 11, 13–19], along with jump height [20], and the modified reactive strength index (RSImod) [21]. Increased asymmetry has been found in ACLR athletes in the eccentric braking rate of force development (RFD), peak force and impulse [3, 5, 10, 11, 13, 14, 17, 22], with recommendations to reduce between-limb kinetic asymmetries below 10% prior to a return to sport [6, 8, 23, 24]. However, the potential for uninvolved limb detraining after ACLR poses a major limitation of the between-limb asymmetry index [23, 25, 26] highlighting the importance of obtaining pre-injury testing to establish neuromuscular benchmarks for CMJ mechanical testing.

An additional limitation of assessing CMJ mechanics with force plates is the analysis of only discrete time points or jump phases, which neglects other regions of the CMJ force-time waveform that could reveal involved limb force impairment [6, 11, 27], such as the unweighting phase. An alternative approach is to evaluate the entire CMJ force-time waveform [28] with statistical parametric mapping (SPM), a statistical method applied across all points on the CMJ force-time tracing [20, 21, 27, 29, 30] offering a more in depth and thorough assessment of overall movement quality [28]. SPM accounts for the magnitude of differences between two curves at every time point and can visually display areas within the time series where significant between-limb differences in force occur that may be overlooked by discrete phase or time point analysis [28, 31, 32]. SPM has been used to examine muscle activation patterns [33], ground reaction forces (GRFs) [34] and joint kinetics and kinematics [31] in injured and noninjured populations and to assess interlimb kinetic asymmetries post ACLR [31, 34]. Applying SPM analysis on pre-injury and post-injury CMJ force-time tracings from elite athletes in conjunction with a traditional discrete biomechanical analysis can help elucidate regions of the CMJ force-time curve that are most severely impaired after ACL injury. As restoring stretch-shortening-cycle function is critically important for return to sport readiness in athletes [35], a complete analysis of the CMJ force-time curve can help practitioners pinpoint specific regions of force loss that can augment rehabilitation and post-injury strength training.

The aim of this study, therefore, was to apply SPM analysis and a traditional biomechanical analysis using discrete jump phases to the CMJ force-time data obtained from elite athletes before and after ACL injury. We hypothesised that (1) both the SPM and traditional approach would reveal reduced involved limb force production in the CMJ propulsive phase; (2) the pre-injury benchmark of the involved limb would reveal CMJ force deficits not seen with the post-ACLR between-limb benchmark and (3) SPM analysis would uncover additional force deficits not detected with the traditional analysis approach.

## 2. Materials and Methods

### 2.1. Design

Longitudinal CMJ testing was conducted with elite athletes post-ACLR (*n* = 20; females: *n* = 8) over four years [36]. Elite athletes were defined as being within ∼7% of world record/leading performance [37]. In order to control for the effects of time on post-injury CMJ force-time asymmetries, 10 athletes with post-injury testing at six months post-surgery (24 ± 3 weeks) were identified and subsequently included in the present analysis (male *n* = 4, female *n* = 6, age = 22.0 ± 3.5 years, mass = 75.9 ± 11.5 kg). Athletes participated in alpine skiing (*n* = 4), freestyle skiing (*n* = 1), luge (*n* = 1), ski cross (*n* = 3) and wrestling (*n* = 1) and performed CMJ testing as a component of an individualised rehabilitation programme conducted by trained professionals. Based on a previous study examining CMJ kinetic asymmetries of elite athletes with ACL injury, a minimum of 9 participants was required to achieve sufficient statistical power (*β* = 20%) [10].

Athletes were included if they sustained a primary ACL rupture with subsequent ACLR (semitendinosus grafts: *n* = 9, bone-patellar-tendon-bone grafts: *n* = 1). Athletes who had sustained secondary injury to other knee structures such as meniscus injury, articular cartilage and medial collateral ligament injury (MCL) were also included. Athletes with severe multiligament injuries were excluded. Athletes were also excluded if they had other lower limb or spine injuries that limited maximal effort CMJ testing. Participants followed an individualised, progressive rehabilitation programme administered by qualified practitioners at the same training centre that combined time and outcome measure–based rehabilitation milestones. This study was approved by the Conjoint Research Ethics Board at the University of Calgary (REB14-2270 REN6) and the Faculty of Health Sciences Research Ethics Committee of the University of Pretoria (380/2021). Participants were aware of the benefits and risks associated with maximal neuromuscular testing and gave written informed consent to participate in the study prior to testing.

### 2.2. Test Procedures

The pre-ACLR baseline test (T0) was defined as the most recent test prior to injury up to a maximum of six months (24.8 ± 21.6 weeks) before surgery, and the post-ACLR test was conducted at 24 ± 3 weeks after surgery (T1). All athletes were familiarised with the testing protocol and regularly performed maximal effort CMJs as a part of off-season training. Testing was supervised by certified strength and conditioning specialists. Participants performed a standardised 10 min warm up on a cycle ergometer and light lower body dynamic stretching before the CMJ test. Dynamic stretches were repeated for 10 repetitions. These stretches focussed on the muscles of the lower limb (i.e., quadriceps, hamstrings, gluteal muscles, hip flexors and plantar flexors). Athletes were instructed to place each foot on two adjacent force plates and remain still to collect a stationary baseline force. Athletes then performed between 5 and 10 maximal effort CMJs during which they were instructed to maximise their vertical jump height using a self-selected squat depth while keeping their hands firmly planted on their hips [10]. CMJ trials that deviated from the instructed technique were discarded and then repeated. A strong verbal cue was provided before each jump to ensure a maximal effort was given.

### 2.3. Force Plate Data Processing

A dual force plate system was used to simultaneously measure left and right vertical GRFs, sampling at 1500 Hz (AMTI Accupower) and gathered using commercial software (Noraxon MR3.14) [10]. Data were recorded on a personal computer and the force-time data were exported to csv files. Test dates that aligned with the pre- and post-ACLR time periods were analysed using the Shiny Vertical Jump Analysis app (https://github.com/mattsams89/shiny-vertical-jump) in RStudio.

The force-time curves of all jump trials were visually inspected for errors, such as absence of a stationary weighing period or minimum force decreasing below 20 N (total unweighting), in which case the trials were excluded. The initiation of the CMJ unweighting phase was initially established using two methods because of the importance of (i) between-participant consistency in the start and end points of time-normalised data for SPM analysis and (ii) the assumption of a stationary start for impulse-momentum calculations. First, after determining body weight (BW) that was averaged over a 1 s quiet standing period, the CMJ initiation was identified as the point at which GRF decreased to BW − 5SD [29, 30]. The second method employed an algorithm that searched backwards from the BW − 5SD point to check if the inverse (BW + 5SD) occurred within the previous 100 ms. This algorithm worked back until the last point before this inverse threshold was broken in order to find the true initiation of movement (i.e., accounting for the potential of a small rise in the body centre of mass before the initiation of the unweighting phase, termed ‘preload'). Method one (BW − 5 SD) was used to analyse all of the time normalised force-time waveform data for the SPM analysis to standardise the detection of the initial downward displacement of the COM. This method excluded minor fluctuations in GRF prior to this point, such that the start and end of the time normalised data were aligned for all participants. Force-time data were normalised to 101 data points for the SPM analysis. Method two (BW + 5SD) was used for the kinetic and asymmetry analysis of the CMJ force-time variables and ensured that the assumption of an initial COM velocity equal to zero, which is required for analysis using the impulse-momentum relationship, was valid. Take-off was determined at the point where GRF decreased below 20 N.

Unweighting, braking and propulsion phases were determined according to previously described methods [30]. Net impulse was calculated as the resultant impulse after removing the effect of gravity on the mass of the athlete's body, through time-integration of the total force-time curve (i.e., left + right GRF). Net peak force (N/kg) and net impulse (N∙s/kg) of the involved and uninvolved limb were calculated for each of these three phases. The impulse-momentum method was used to calculate take-off velocity and subsequently jump height, and the trial with the greatest jump height was used for the analysis.

An asymmetry index [38] was then calculated and reported as a percentage, using the following formula:

(1) $$\begin{array}{c} \frac{\left(\text{Uninvolved limb}-\text{involved limb}\right)}{\left(\text{Sum of uninvolved and involved limbs}\right)}\times 100. \end{array}$$

In addition to force and impulse metrics, discrete CMJ outcome measures commonly used in applied settings were reported for further context. These measures included jump height (m), contraction time (s), RSImod (AU) and the total peak force (N/kg). Contraction time was calculated as the difference between take-off and the initiation of movement (utilising method 2 to determine initiation of movement as BW + 5SD). Total force (*F*_TOTAL_) was calculated as the sum of the right and left net peak vertical GRFs. RSImod was calculated as the ratio of jump height to contraction time.

### 2.4. Statistical Analysis

Descriptive statistics (mean ± SD) for all discrete performance variables were calculated for T0 and T1. Comparisons between the test time points were performed using paired sample *t*-tests after confirming that the data were normally distributed using the Shapiro–Wilk test. Alternatively, the Wilcoxon rank test was applied if there was a non-normal distribution. Time normalised continuous force-time data were analysed using SPM, which calculated a single test statistic for each data point of the CMJ compared to a critical threshold. Jump height, contraction time, RSImod and the F_TOTAL_ were only compared between post-injury (T1) and pre-injury (T0) testing time points. Each limb was then compared to itself for pre-injury and post-injury measurements, whereas the involved limb was compared to the uninvolved limb at the pre-injury testing time point (T0) and post-injury testing time point (T1). Statistical significance for SPM and the other tests was set at *p*  <  0.05.

## 3. Results

### 3.1. Traditional Biomechanical Analysis

No statistically significant differences in CMJ performance measures (jump height, contraction time or RSImod) were found, but F_TOTAL_ (*p* = 0.009) (Table 1) and the involved limb peak force were lower post-ACLR compared to pre-injury (*p* = 0.002) (Table 2). Greater asymmetry in peak force was also found at T1 (7.3 ± 11.1%) compared to T0 (−1.1 ± 6.1%) (*p* = 0.039) (Table 2), reflecting reduced involved limb force.

Lower unweighting impulse was found for the involved limb compared to the uninvolved limb pre-injury (*p* = 0.012) (Table 1), leading to a higher unweighting impulse asymmetry pre-injury compared to post-ACLR (*p* = 0.017) (Table 2). Lower propulsive impulse production was found for the involved limb compared to the uninvolved limb post-ACLR (*p* = 0.007) (Table 2), which corresponded to a higher propulsive asymmetry index when comparing T1 post-ACLR to T0 pre-ACLR (*p* = 0.011) (Table 1). Finally, the uninvolved limb showed a greater propulsive impulse after ACLR compared to itself pre-injury (*p* = 0.037) (Table 2).

### 3.2. SPM Force-Time Waveform Comparisons

**Figure 1:**
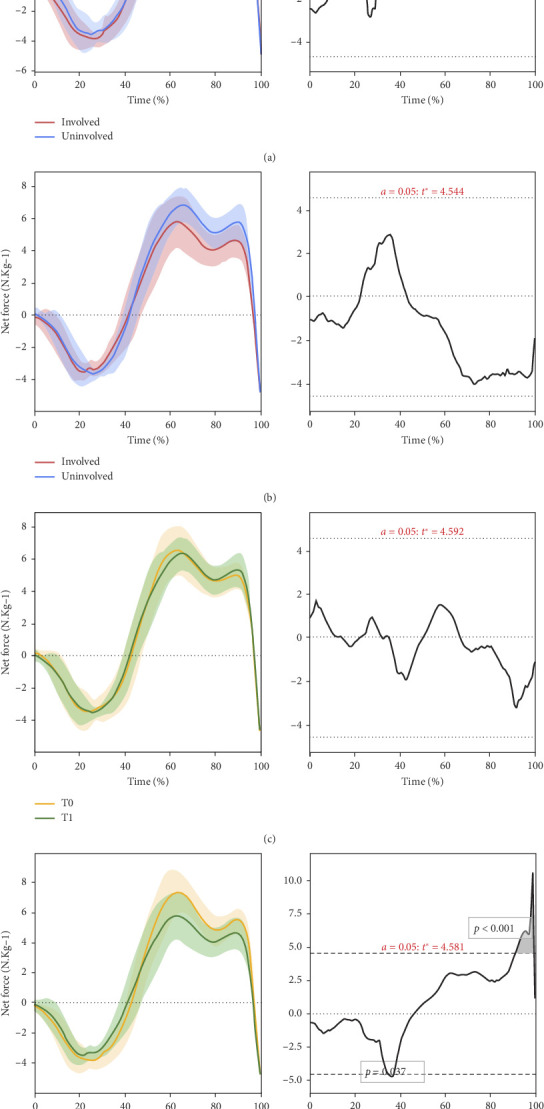
Statistical parametric mapping analysis of the countermovement jump force-time waveform from movement initiation (0%) to take-off (100%) (left column). Figures in the right column indicate where the critical threshold for significance (dashed lines) is broken for the statistical parametric mapping test statistic. ⁣^∗^A statistically significant difference (alpha < 0.05) where the critical test threshold (*t*) was exceeded. (a) Uninvolved limb compared to the involved limb at T0. (b) Uninvolved limb compared to the involved limb at T1. (c) Involved limb compared to itself at T0 versus T1⁣^∗^. (d) Uninvolved limb compared to itself at T0 versus T1.

No statistical between-limb differences were found at baseline before injury (Figure 1(a)). Additionally, while the involved limb force production was reduced compared to the uninvolved limb at T1 post-ACLR, it did not reach statistical significance (Figure 1(b)).

Involved limb force production decreased post-ACLR compared to pre-ACLR (i.e., within-limb comparison from T1 to T0) from 36% to 37% (*p* = 0.037) and 92%–99% (*p* < 0.001) of the CMJ waveform (Figure 1(c)). These two periods coincided with the latter half of the unweighting phase (downwards acceleration was decreasing) and the end of the propulsion phase. The uninvolved limb showed no within-limb force production changes when comparing pre-injury and post-injury CMJ force-time curves (Figure 1(d)).

## 4. Discussion

This study provides new perspectives on the best analytical approach to evaluate CMJ mechanics before and after ACL injury in elite athletes. While the sample size was small (*n* = 10), previous work suggests we had sufficient statistical power to evaluate the primary hypotheses [10] and we prioritised controlling for the effects of the post-ACLR time interval on the CMJ force loss requiring us to reduce the sample size from 20 to 10 participants. Nevertheless, the opportunity to collect longitudinal data (including pre-injury control data) in elite and world class level athletes who eventually went on to sustain ACL injury contributes important perspectives to the scientific literature on post-ACLR testing methodologies. Consistent with our hypotheses, propulsive phase force deficits were found for the involved limb compared to the uninvolved limb with both the traditional biomechanical analysis and the SPM waveform analysis. However, the pre-injury baseline comparisons revealed important within-limb force loss of the involved limb that was not detectable through the post-injury between-limb comparison. Finally, in support of our third hypothesis, SPM analysis revealed statistical differences in the within-limb force decrements of the involved limb from baseline to post-ACLR, corresponding to reduced propulsive phase force and decreased force production in the late unweighting to braking phase transition of the CMJ.

These results highlight that SPM analysis may provide complementary information alongside discrete phase and time point analysis when it comes to detecting involved limb force deficits after ACLR. Notably, while a decrease in involved limb peak force production was found from baseline to post-ACLR using the discrete time point analysis, SPM also made visible that the involved limb force output had reduced with respect to transition between the unweighting and braking phases and the late propulsive phase. No differences in the involved limb unweighting impulse (*p* = 0.160) and braking impulse (*p* = 0.587) were found from baseline to post-injury. Further, it should be noted that the region showing a statistical difference with SPM bridged the unweighting and braking phases, which may be why the discrete phase analysis failed to reveal a loss of force production since it considered unweighting and braking as separate phases.

Altogether, the regions showing a statistical difference in involved limb force production with the SPM analysis corresponded to about 10% of the total CMJ waveform. But more interestingly, the fact SPM analysis revealed altered force output of the involved limb in the transition between the unweighting and braking phases may be critically relevant for restoring vertical jump capacity after ACL injury since this phase may be crucial for promoting an effective CMJ strategy [39]. Therefore, we suggest that clinicians and practitioners should also consider evaluating the unweighting phase and braking phase transition after ACL injury when evaluating the involved limb compared to itself in order to address the potential for force loss.

Analysis of discrete kinetic variables is often used to assess jump performance [40], but in the case of post-ACLR testing, a more agnostic inspection of the entire force-time curve can assist practitioners in detecting additional aspects of the CMJ force-time curve that are affected by ACL injury that may not be readily visible or obscured by such analysis. SPM analysis revealed differences that were benchmark-dependent relating to the CMJ movement strategy, notably reduced unweighting, and propulsive force of the injured limb at T1 compared to itself at T0. A majority of the literature examining neuromuscular function after ACLR has used the post-injury contralateral limb benchmark for asymmetry calculations [3, 5, 10, 11, 13–15, 17], while the incorporation of pre-injury benchmarks is more scarce [25, 26]. The typical use of the post-injury contralateral limb benchmark may overestimate an athlete's rehabilitation status and return to sport readiness due to the potential detraining of the uninvolved limb [25].

Based on our results and previous findings, practitioners may consider prioritising a pre-injury benchmark of the involved limb, when possible, to monitor jump kinetics and force-time data during CMJ mechanical testing before returning to sport. Although our current study did not show detraining of the uninvolved limb, it has been pointed out in previous research and should therefore be a consideration when assessing interlimb asymmetries after ACLR [25]. Examining the force-time curve on an individual basis with waveform analysis may also assist in identifying specific regions of force loss in ACLR athletes. This may be used to better inform rehabilitative programs and guide return to play decision making and monitoring.

## 5. Perspective

Using pre- and post-injury data from a group of elite athlete participants who sustained ACL injury, we showed that analysis of discrete CMJ kinetic variables and SPM of the force-time waveform revealed reductions in involved limb force production after ACL surgery. However, differences in analysis methods were noted in that only SPM analysis showed reduced propulsive phase force and decreased force production for the involved limb (T1) compared to itself pre-injury (T0) in the late unweighting to braking phase transition of the CMJ. This highlights the potential advantages of a combined analysis approach and consideration for using pre-injury limb force production to identify post-ACLR strength deficits in an elite athlete population.

## 6. Limitations

This study employed a small sample size but included elite world class athletes to examine CMJ force-time differences before and after ACLR. Consequently, our results may not apply to nonelite athletes and a larger sample size could further increase the chance of finding statistically different force decrements with a smaller effect size. Also, participants were not sex and age matched. However, these results may still provide clinically relevant information on the CMJ mechanics of elite athletes as force deficits were found for the involved limb during crucial functional phases of the CMJ that contribute to effective stretch-shortening-cycle function. The inclusion of elite and world class level athletes and the use of pre-injury control data as a reference point provide compelling insights into post-ACLR force production deficits within this population based on the application of a standard kinetic analysis combined with SPM.

## Figures and Tables

**Table 1:** Countermovement jump performance metrics pre- (T0) and post-surgery (T1).

	Jump height (m)	Contraction time (s)	RSImod (AU)	*F *_TOTAL_ (N/kg)
T0	0.37 ± 0.05	0.84 ± 0.15	0.44 ± 0.08	15.0 ± 2.3
T1	0.35 ± 0.09	0.89 ± 0.08	0.39 ± 0.09	**13.6 ± 2.0 **⁣^∗^
*p* value	0.084	0.358	0.178	0.009

*Note:* Values are presented as mean ± standard deviation with statistically significant differences in bold font. *F*_TOTAL_, total combined (left and right limb) peak force.

Abbreviations: AU, arbitrary units; RSImod, reactive strength index modified.

⁣^∗^Difference between T0 and T1.

**Table 2:** Countermovement jump involved and uninvolved limb force, impulse and asymmetry indices pre- (T0) and post-surgery (T1).

	Peak force (N/kg)			Impulse (N·s/kg)								
				Unweighting phase			Braking phase			Propulsion phase		
	T0	T1	*p* value	T0	T1	*p* value	T0	T1	*p* value	T0	T1	*p* value
Involved	7.7 ± 1.4	6.4 ± 1.6^**t**^	0.002	−0.82 ± 0.13	−0.83 ± 0.41	0.160	0.74 ± 0.15	0.70 ± 0.26	0.587	1.34 ± 0.15	1.26 ± 0.54	0.084
Uninvolved	7.5 ± 1.0	7.3 ± 0.7	0.494	−0.67 ± 0.12^**l**^	−0.81 ± 0.40	0.375	0.74 ± 0.09	0.80 ± 0.22	0.695	1.33 ± 0.11	−1.58 ± 0.55^**t**,**l**^	0.037
*p* value	0.468	0.065		0.012	0.746		0.908	0.695		0.784	0.007	
Asymmetry (%)	−1.1 ± 6.1	7.3 ± 11.1^**t**^	0.039	−10.0 ± 10.6	−0.7 ± 10.6^**t**^	0.017	0.2 ± 10.5	7.9 ± 20.6	0.233	−0.2 ± 4.8	−12.3 ± 13.3^**t**^	0.011

*Note:* Values are presented as mean ± standard deviation with statistically significant differences in bold font.

^l^Difference between limbs.

^t^Difference between T0 and T1.

## Data Availability

The research data used for this study are unavailable due to their confidentiality.
