# Correction to “Countermovement Jump Kinetic Impairments in Elite Athletes Before and After ACL Injury: Force-Time Waveform Versus Discrete Kinetic Analysis”

**DOI:** 10.1155/tsm2/9803086

**Published:** 2025-10-03

C. de França, M. J. Jordan, T. Botha, and H. Bayne, “Countermovement Jump Kinetic Impairments in Elite Athletes Before and After ACL Injury: Force-Time Waveform Versus Discrete Kinetic Analysis,” *Translational Sports Medicine* 2025 (2025): 1176787, https://doi.org/10.1155/tsm2/1176787.

In the article titled “Countermovement Jump Kinetic Impairments in Elite Athletes Before and After ACL Injury: Force-Time Waveform Versus Discrete Kinetic Analysis,” there was an error in Figure 1. The figure panels (c) and (d) should be interchanged. The corrected figure is shown below and is listed as Figure 1:

We apologize for this error.

## Figures and Tables

**Figure 1:**
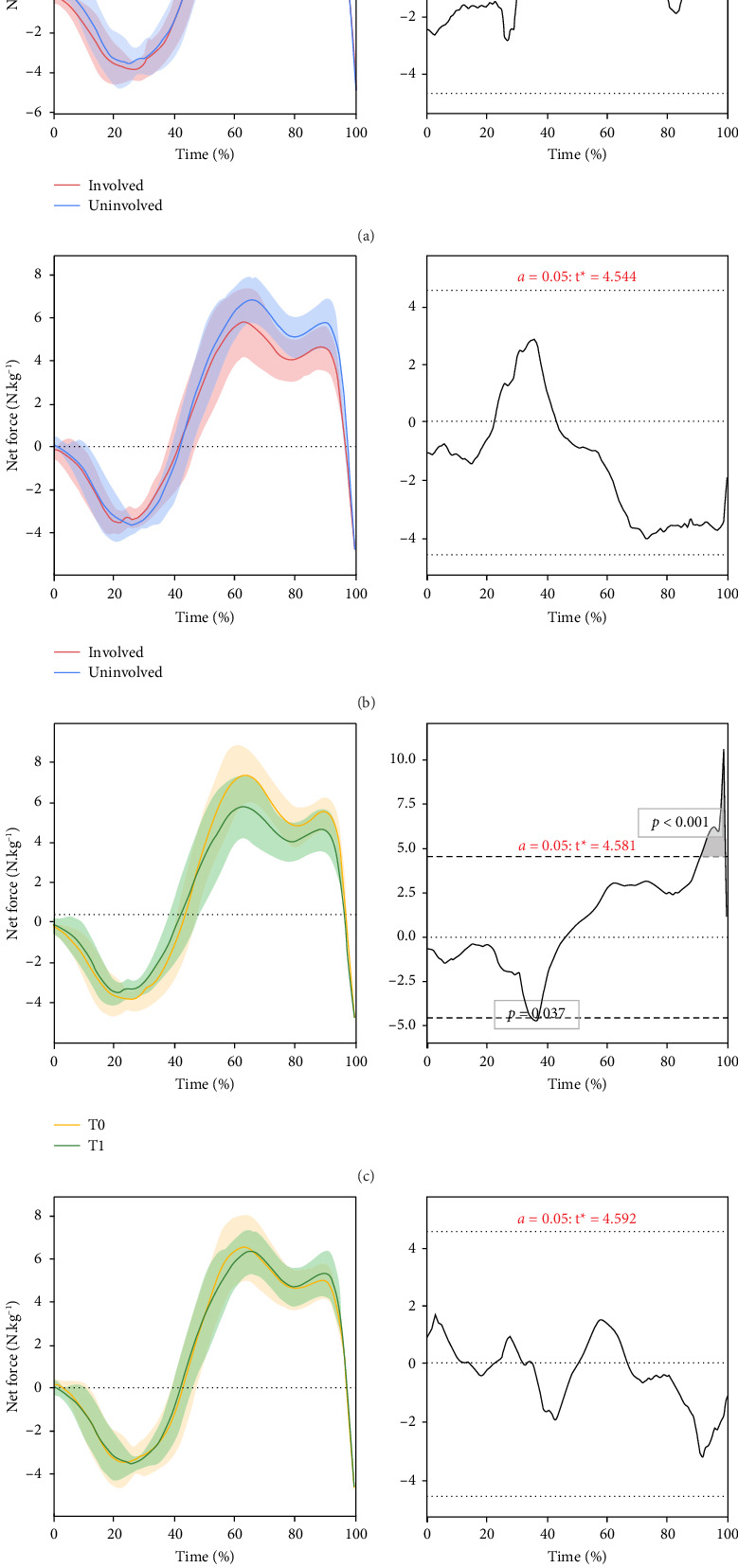
Statistical parametric mapping analysis of the countermovement jump force-time waveform from movement initiation (0%) to take-off (100%) (left column). Figures in the right column indicate where the critical threshold for significance (dashed lines) is broken for the statistical parametric mapping test statistic. ^∗^A statistically significant difference (alpha < 0.05) where the critical test threshold (*t*) was exceeded. (a) Uninvolved limb compared to the involved limb at T0. (b) Uninvolved limb compared to the involved limb at T1. (c) Involved limb compared to itself at T0 versus T1^∗^. (d) Uninvolved limb compared to itself at T0 versus T1.

